# Comment in Response to “Temozolomide in Glioblastoma Therapy: Role of Apoptosis, Senescence and Autophagy etc. by B. Kaina”

**DOI:** 10.3390/biomedicines8040093

**Published:** 2020-04-20

**Authors:** Mike-Andrew Westhoff, Tim Baisch, Verena J. Herbener, Georg Karpel-Massler, Klaus-Michael Debatin, Hannah Strobel

**Affiliations:** 1 Department of Pediatrics and Adolescent Medicine, University Medical Center Ulm, D-89075 Ulm, Germany; tim.baisch@uni-ulm.de (T.B.); verena.herbener@uni-ulm.de (V.J.H.); klaus-michael.debatin@uniklinik-ulm.de (K.-M.D.); hannah.strobel@uniklinik-ulm.de (H.S.); 2 Department of Neurosurgery, University Medical Center Ulm, D-89081 Ulm, Germany; georg.karpel@gmail.com

It is with great pleasure that we acknowledge the fact that our review on Temozolomide (TMZ) has initiated a discussion [1,2,3]. This had been our intent, since a discussion on TMZ is long overdue. Aside from still being the standard chemotherapeutic for Glioblastoma, it is also increasingly being investigated in other contexts; for example, the U.S. National Library of Medicine lists 26 clinical trials for TMZ and Neuroblastoma, the most common extracranial solid tumour in children [4]. Importantly, many of these studies are looking at combinations of drugs; in the case of Neuroblastoma, often TMZ, Irinotecan and something else in addition, in the case of Glioblastoma, there is not only the RIST protocol [5] or the CUSP9* approach [6], but also the presence of TMZ during every Glioblastoma trial, where the standard treatment is compared to the standard treatment plus additions. To optimize these combination therapies, we need to understand exactly not only what the individual components do, but also when they do it, the mode of action and pharmacodynamics, which will be essential for complex therapeutic approaches. The timeliness of this debate is best summarized by Stepanenko and Chekhonin in their conclusion [3]: “… [TMZ]s therapeutic efficiency even in patients with MGMT-methylated tumours is limited, clearly suggesting that alternative or additional therapeutic approaches are urgently needed.” 

In addition to Stepanenko and Chekhonin’s discussion on clinically relevant concentrations of TMZ and the rather disappointing role of MGMT as a predictor for TMZ sensitivity in Glioblastoma [3], we would like to address two further points of difference between Kaina’s analysis of the literature and our own. 

## 1. TMZ as an Apoptosis Inducer

Kaina argues in this response that “apoptosis, autophagy and senescence are therapeutically important endpoints” [2], which we do not dispute and we do apologize if our review gave that impression. In their recent work, Professor Kaina’s group show treatment with TMZ leading to 20% apoptosis and 30% senescence [7]. In our review, we argue that the fact that a cytostatic feature, for example senescence, is found to be consistently more strongly induced than classical apoptosis should lead us to re-evaluate what the primary biological consequences of TMZ exposure are. This argument is supported by the data presented by Aasland and co-workers. Furthermore, this is also consistent with earlier work by Ochs and Kaina, showing that cell death induced by methylating agents generating O6-methylguanine is influenced by MGMT expression, but also that a decline in DNA damage (as assessed by Olive tail moment) precedes cell death, and that cell death can be reduced, but not inhibited, by high concentrations of caspase inhibitors [8]. This data set suggests that even the classical cell death component of TMZ treatment should not be reduced to only apoptosis induced by the presence of double strand DNA breaks.

## 2. The Use of Single High Doses of TMZ as a Surrogate for Repeated Administration of Lower Doses

While many pharmacokinetic studies address the absorption of TMZ into the blood and plasma, its metabolism and excretion via urine in the elderly [9,10,11,12], only a limited amount of studies assessed the neuropharmacokinetics of TMZ in humans, including its penetration into the cerebral spinal fluid [13,14]. Nevertheless, these studies demonstrated that TMZ is characterised by reproducible linear pharmacokinetics and a short half-life, consequently, TMZ does not accumulate after multiple administration [15]. Therefore, the tumour cells are exposed to different concentrations of TMZ over time that, however, do not exceed a specific maximum. Furthermore, it is quite likely that tumour cells at the invading edges are exposed to much lower concentrations of TMZ than cells in the tumour bulk, where microvessels are characterized by an intermediated paracellular permeability [16,17].

Additionally, Stevens and colleagues observed schedule-dependent anti-tumour activity of TMZ in various murine tumour models [18], which was confirmed by several clinical trial reports in glioma patients [11,19,20]. Meanwhile, similar observations had been made in vitro as well. Beier and colleagues compared five different clinically relevant dosing schemes of TMZ in vitro and investigated their effects on clonogenic survival of Glioblastoma stem-like cells (SCs). TMZ-sensitive Glioblastoma SCs responded equally to the 5 days on/23 days off (the Stupp protocol), 21 days on/7 days off, 7 days on/7 days off regimens, though to a greater extend compared to single dose TMZ at high concentrations. Differences in the induction of cell death, however, were not observed between these dosing schemes [21]. These findings question the use of a single dose of TMZ as a surrogate, as it is performed in many in vitro studies, and further studies are definitely required to analyse the influence of the dosing scheme on the biological effect observed in vivo. Lessons from radiotherapy have already taught us that a single dose of irradiation poorly reflects fractionated radiation therapy in GB cells [22].
